# Targeting the WNK-SPAK/OSR1 Pathway and Cation-Chloride Cotransporters for the Therapy of Stroke

**DOI:** 10.3390/ijms22031232

**Published:** 2021-01-27

**Authors:** Sunday Solomon Josiah, Nur Farah Meor Azlan, Jinwei Zhang

**Affiliations:** Hatherly Laboratories, Institute of Biomedical and Clinical Sciences, Medical School, College of Medicine and Health, University of Exeter, Exeter EX4 4PS, UK; josiahsos2014@gmail.com (S.S.J.); nm503@exeter.ac.uk (N.F.M.A.)

**Keywords:** stroke, electroneutral transport, cation-chloride cotransporters, KCCs, NKCCs, WNK-SPAK/OSR1

## Abstract

Stroke is one of the major culprits responsible for morbidity and mortality worldwide, and the currently available pharmacological strategies to combat this global disease are scanty. Cation-chloride cotransporters (CCCs) are expressed in several tissues (including neurons) and extensively contribute to the maintenance of numerous physiological functions including chloride homeostasis. Previous studies have implicated two CCCs, the Na^+^-K^+^-Cl^−^ and K^+^-Cl^−^ cotransporters (NKCCs and KCCs) in stroke episodes along with their upstream regulators, the with-no-lysine kinase (WNKs) family and STE20/SPS1-related proline/alanine rich kinase (SPAK) or oxidative stress response kinase (OSR1) via a signaling pathway. As the WNK-SPAK/OSR1 pathway reciprocally regulates NKCC and KCC, a growing body of evidence implicates over-activation and altered expression of NKCC1 in stroke pathology whilst stimulation of KCC3 during and even after a stroke event is neuroprotective. Both inhibition of NKCC1 and activation of KCC3 exert neuroprotection through reduction in intracellular chloride levels and thus could be a novel therapeutic strategy. Hence, this review summarizes the current understanding of functional regulations of the CCCs implicated in stroke with particular focus on NKCC1, KCC3, and WNK-SPAK/OSR1 signaling and discusses the current and potential pharmacological treatments for stroke.

## 1. Introduction of Cation-Chloride Cotransporter Family

The family of cation-chloride cotransporters (CCCs) comprises the Na^+^-K^+^-Cl^−^, Na^+^-Cl^−^, and K^+^-Cl^−^ cotransporters (NKCCs, NCC, and KCCs). Identification of these CCCs in several tissues such as red blood cells, epithelia, and neurons have alluded to their extensive contributions to ion and water homeostasis, both at a cellular and trans-epithelial level [1,2,3]. The identification of the functional properties of most of these transporters dates back to the late 1970s and early 1980s as Cl^−^-dependent cation fluxes, with red blood cells and Ehrlich ascites tumor cells constituting pivotal model tissues [4,5,6,7]. Subsequently, their molecular identities were established about a decade afterwards [8,9,10]. CCCs are intrinsic membrane proteins that move Na^+^, K^+^, and Cl^−^ ions across plasma membranes in a tightly coupled electroneutral manner. They facilitate secondary active transport driven by the gradients generated by the Na^+^/K^+^-ATPase [11]. The solute carrier family 12 (SLC12) of the CCC family consists of nine members [2]. A group of three Na^+^-dependent inward cotransporters comprises of one Na^+^-Cl^−^ cotransporter (NCC)—its sole isoform is found in the kidney and encoded by *SLC12A3* [9] and two Na^+^-K^+^-Cl^−^ cotransporters isoforms (NKCC1 and 2)—NKCC1 is ubiquitous whilst NKCC2 is specifically expressed in the kidney and are encoded by *SLC12A2* and *SLC12A1*, respectively [2]. Na^+^-independent outward transport of K^+^ and Cl^−^ is facilitated by four K^+^-Cl^−^ cotransporters with distinct functional properties (KCC1 [12], KCC2 [13], KCC3, and KCC4 [14,15]). The KCC isoforms are encoded by *SLC12A4–7* respectively, of which *SLC12A5* (KCC2) is found exclusively in neurons [2]. The additional SLC12 family members, CCC9 and CCC-interacting protein (CIP), are encoded by *SLC12A8* and *SLC12A9,* respectively, and have no physiological role ascribed to them yet [16], though recent genome-wide association studies found novel *SLC12A8* variants may be associated with dyslipidemia [17], and *SLC12A9* may be involved in feather pecking and aggressive behavior [18] (see Table 1).

All proteins in the CCC family have common functional characteristics. These include (1) the coupled transport of one cation (Na^+^ and/or K^+^) per individually transported anion, hence the appellation of electroneutral cotransporters, (2) chloride is always the transported anion, (3) all cotransporters are modulated by variations in cell volume, (4) changes in the intracellular chloride concentration ([Cl^−^]_i_) influence the modulation of their expression, and (5) the regulation of CCCs activity is achieved through phosphorylation and dephosphorylation processes [19]. The functional and structural characteristics of the Na^+^ dependent and Na^+^ independent branches clearly distinguish the two. The degree of identity amongst the Na^+^ dependent transporters and the Na^+^ independent are 50% and 70% respectively. Between the two NKCC isoforms, the degree of identity is 25% [20].

Stroke is one of the major culprits responsible for global death and disability [21]. Currently, there is a paucity of pharmacological strategies to reduce the mental damage as well as the burden triggered by this pathology. Ischemic stroke is the most common type of stroke, which accounts for approximately 85% of the cases of the pathology [22]. Ischemia is the disruption of blood flow and the subsequent depletion of oxygen and glucose. As neuronal components strictly function on aerobic metabolism [23], an ischemia in the brain leads to reduced available ATP levels and ionic imbalance across the neuronal cell membrane [24], which causes irreversible neuronal death, also known as ischemic stroke [25,26,27]. During this process, an imbalance of excitatory glutamate and inhibitory gamma amino acid butyric acid (GABA) further accelerate neuronal demise [28,29,30], subsequently leading to the onset of post-stroke seizures [20,31,32]. Neuronal cells excitation is opposed by inhibitory GABA through the activation of GABA_A_ receptors. The activation of the GABA_A_ receptors is dependent on the chloride transmembrane gradient [23]. Notably, CCCs are the primary regulators of chloride homeostasis in the brain [2,33]. This role is accomplished through the extrusion of Cl^−^ via KCC2 and entry of Cl^−^ via NKCC1 which regulates [Cl^−^]_i_. GABA is inhibitory as a result of lower [Cl^−^]_I_, driven by higher expression of KCC2. Interestingly, immature neurons express less KCC2 and more NKCC1 leading to a higher [Cl^−^]_i_ and excitatory GABA. The switch from excitatory to inhibitory during neurodevelopment, a process termed excitatory-to-inhibitory GABA switch, is generated through reduction in NKCC1 level and increase in KCC2 level [34]. The expanding work on CCC influence on neuronal excitability in physiological conditions especially during development and pathological conditions suggest that they could be a new treatment approach for stroke [27].

In view of this, constant updates on the role of CCCs in stroke and their regulation is highly germane for the development of therapeutic drugs in the management of this pathology. Thus, the aim of this review is to summarize the current understanding of functional regulation of the CCCs and particularly the role of NKCC1 and KCC3 cotransporters in the pathogenesis of stroke. Then, the regulatory role of the with-no-lysine kinase (WNKs) family and STE20/SPS1-related proline/alanine rich kinase (SPAK) or oxidative stress response kinase (OSR1) (WNK-SPAK/OSR1) signaling pathway in stroke will be considered. Lastly, current pharmacological treatments for stroke with respect to potent inhibitors of WNK-SPAK/OSR1 pathway and NKCC1 cotransporter, and activators of KCC3 transporter will be discussed in this review.

**Table 1:** The solute carrier family 12 (SLC12) of cation-chloride cotransporters in neurological disorders and others. TAL: thick ascending loop of Henle; DCT: distal convoluted tubule; RVI: regulatory volume increase; RVD: regulatory volume decrease; ND: no data (or none). Functional regulation of the cation-chloride cotransporter family.

Encoding Gene (Protein)	Co-Transport Ions	Tissue Distribution	Physiological Functions	Genetic Disorders	References
*SLC12A1* (NKCC2)	Na^+^, K^+^, Cl^−^	Kidney-specific (TAL)	NaCl reabsorption in the TAL; regulation of Ca^2+^ excretion; urine concentration	Bartter’s syndrome	[2,35,36,37]
*SLC12A2* (NKCC1)	Na^+^, K^+^, Cl^−^	Ubiquitous	Cell volume regulation (RVI); provide ions for secretion	Potential role in human schizophrenia multi-organ system failure, congenital hydrocephalus, hearing, and neurodevelopmental disorder	[2,38,39,40,41,42,43]
*SLC12A3* (NCC)	Na^+^, Cl^−^	Kidney-specific (DCT)	NaCl reabsorption in the DCT; regulation of Ca^2+^ and K^+^ renal excretion;	Gitelman’s syndrome	[9,44,45]
*SLC12A4* (KCC1)	K^+^, Cl^−^	Ubiquitous	cell volume regulation (RVD), KCl epithelial Transport	ND	[2,12,45,46]
*SLC12A5* (KCC2)	K^+^, Cl^−^	Neuron-specific	Intraneuronal Cl^−^ Concentration regulation	Idiopathic generalized epilepsy, developmental apoptosis, neurodevelopmental pathology, Rett syndrome	[2,13,47,48,49,50,51]
*SLC12A6* (KCC3)	K^+^, Cl^−^	Widespread	Volume regulation in the brain; K^+^ recycling in the kidney	Anderman’s syndrome, Charcot–Marie–Tooth disease, hydrocephalus, sensorimotor neuropathy	[2,14,15,52,53,54,55,56,57]
*SLC12A7* (KCC4)	K^+^, Cl^−^	Widespread	Participates in acid excretion in alpha intercalated cells of collecting duct	ND	[2,15]
*SLC12A8* (CCC9)	Unknown	Widespread	No function ascribed yet	Psoriasis, dyslipidemia	[2,16,17,58,59]
*SLC12A9* (CIP)	Unknown	Widespread	No function ascribed yet	May be involved in feather pecking and aggressive behavior	[2,16,18,58]

**Figure 1:**
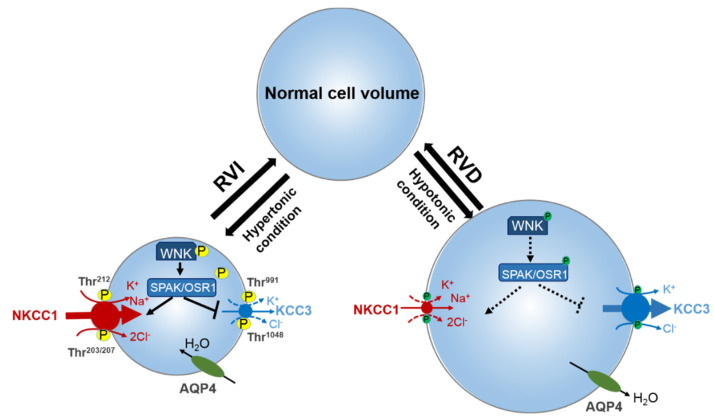
Roles of cation-chloride cotransporters (CCCs) in cell osmoregulation. Intracellular osmolarity changes activate cellular volume regulation. Under hypertonic extracellular conditions of cell shrinkage due to water extrusion from the cell, a counter-response of regulatory volume increase (RVI) restores normal cell volume. In this condition, the WNK-SPAK/OSR1 pathway is activated leading to the phosphorylation of the CCCs. This activates NKCC1 and inhibits KCC, leading to the NKCC1-mediated influx of Na^+^, K^+^, and Cl^−^ along with water, thus restoring cell volume. On the contrary, under hypotonic stress conditions of cell swelling, the cell activates a regulatory volume decrease (RVD). The WNK-SPAK/OSR1 pathway remains inactive and NKCC1 and KCCs are dephosphorylated. This stimulates KCC3 but inhibits NKCC1 leading to the efflux K^+^ and Cl^−^ along with water, and cell volume decrease. NKCC1, K^+^-Cl^−^ cotransporters; KCC3, K^+^-Cl^−^ cotransporter 3; WNK, with-no-lysine kinase; SPAK, STE20/SPS1-related proline/alanine rich kinase; OSR1, oxidative stress response kinase; AQP4, aquaporin. Part of figure elements were adapted from Huang et al. [65].

Undoubtedly, for a cell to function properly it is essential to maintain constant intracellular ionic milieu [53]. Homeostasis of [Cl^−^]_i_ in particular, influences the movement of fluid across epithelia, the polarity of GABA, and more. The electroneutral CCCs are critical determinants of [Cl^−^]_i_ [53,60]. The [Cl^−^]_i_ gradient across the neuronal membrane is crucial for controlling the polarity of GABAergic signaling. GABA_A_ conducts Cl^−^ ions. The direction of Cl^−^ movement through GABA_A_, which determines whether it is excitatory or inhibitory, is dependent on the [Cl^−^]_i_ gradient. Entry of Cl^−^ through GABA_A_ results in the hyperpolarization of neurons and the extrusions of Cl^−^ through GABA_A_ depolarizes the neurons [23,61,62,63]. Changes in expression levels of the CCCs during development reverses the chloride gradient in neurons, generating a switch from an excitatory GABA to an inhibitory GABA [23,64]. NKCCs facilitate Cl^−^ movement into the cell, while KCCs facilitates Cl^−^ movement out of the cell. Thus, NKCCs promote an increased participation of [Cl^−^]_i_ in the pathways for regulatory volume increase (RVI), while the KCCs promote decrease in the [Cl^−^]_i_ as one of the regulatory volume decrease (RVD) mechanisms [19,53,65] (Figure 1). These evolutionarily conserved transporters are amongst the most important mediators of ion transport in multicellular organisms, with particular importance in mammalian central nervous system (CNS) regulation of ionic and water homeostasis [66]. As mentioned earlier, the CCCs are involved in several important cellular functions such as trans-epithelial ion transport, cell volume regulation, and maintenance of [Cl^−^]_i_. Their importance in physiological function is evident by the many human Mendelian disorders of the brain and renal phenotype that arise due to mutations in some members of the CCC family and their upstream regulators [19] (Table 1). For instance, reduction in neuronal KCC2 activity results in decreased inhibition and a hyper-excitable network, a feature shared amongst numerous neurological disorders including epilepsy, autism, post-surgical complication, neuropathic pain, and neuropsychiatric disorders [48,53,67,68].

Since CCCs are key players in several important cellular functions and principally responsible for reciprocal cations (Na^+^ and K^+^) exchange with Cl^−^ to maintain cellular balance, regulatory mechanisms are crucial to coordinate their activity [53,60,67,69]. Indeed, several kinases and phosphatases regulate their transport activity. However, previous reports have established that members of the WNKs family and their downstream targets, SPAK and OSR1, are the master regulators of CCCs activity [1,53,60,65,70,71,72,73,74,75].

**Figure 2:**
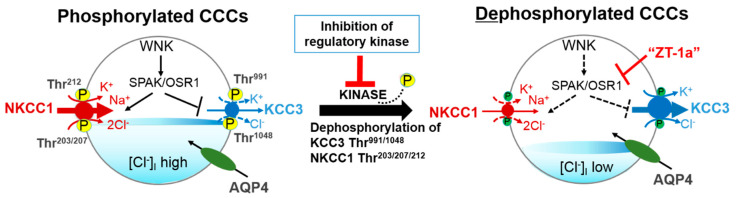
A novel strategy to facilitate cellular Cl^−^ extrusion by coincident NKCC1 inhibition and KCC3 activation by inhibiting Table 1 kinases. Reversible serine-threonine phosphorylation reciprocally regulates NKCC1 and KCC3. Hypotonic low [Cl^−^]_i_ conditions or a reduction in cell volume activates the WNK-SPAK/OSR1 pathway to promote Cl^−^ and water influx. This leads to the phosphorylation of NKCC1 and KCC3 and their activation and inhibition respectively. When [Cl^−^]_i_ becomes too high or cell volume increases, WNK-SPAK/OSR1 pathway is inhibited. The cotransporters are dephosphorylated, KCC3 is activated and facilitates [Cl^−^]_i_ and water efflux to restore ion and osmotic homeostasis. CCCs, cation-chloride cotransporters; NKCC1, K^+^-Cl^−^ cotransporters; KCC3, K^+^-Cl^−^ cotransporter 3; WNK, with-no-lysine kinase; SPAK, STE20/SPS1-related proline/alanine rich kinase; OSR1, oxidative stress response kinase; AQP4, aquaporin 4; ZT-1a, specific SPAK inhibitor. Part of figure elements were adapted from Salihu et al. [85].

Though it has been appreciated for some three decades that protein phosphorylation coupled with external osmotic environment are crucial components in regulation of CCC activities [2,54,76,77,78,79,80], knowledge on the enzymes that regulate these signaling networks was sparse then. The WNK family encoded by the genes *WNK1–4* [81], *SPAK*, and *OSR1* play crucial roles in the regulation of cell volume homeostasis through the regulation of intracellular Na^+^, K^+^, and Cl^−^ [53,82]. The many roles of the WNK-SPAK/OSR1-CCC pathway which include cell volume homeostasis, epithelial transport, and GABA signaling are associated with an array of pathologies which include essential hypertension, cerebral edema, anemia, and neuropathic pain [1,19,53,60,65,67,71,73]. In response to osmotic stress of low [Cl^−^]_i_, isoforms of WNK are activated through phosphorylation. The WNK isoforms then phosphorylate the related downstream kinases SPAK and/or OSR1 [83,84]. Activated SPAK and/or OSR1 phosphorylates the CCCs, which activates NCC, NKCC1, and NKCC2 but inhibits KCCs through a reciprocal regulatory mechanism [67,72] (Figure 2). The counter regulation of the CCCs coordinates Cl^−^ movement across the membrane to maintain Cl^−^ homeostasis and circumvent superfluous energy utilization [65].

**Figure 3:**
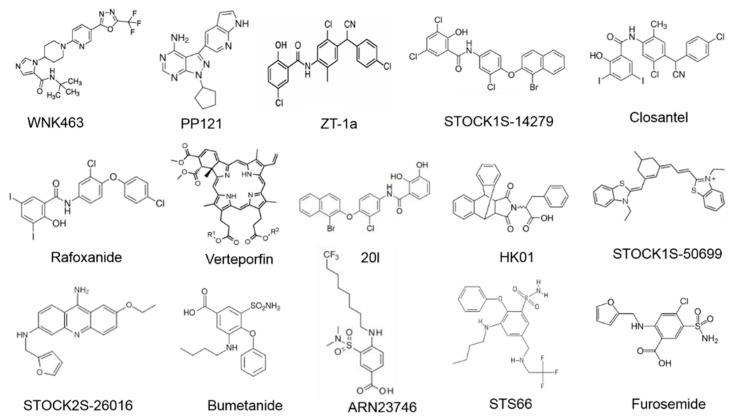
Molecular structures of WNK-SPAK/OSR1-CCCs signaling pathway compounds.

**Figure 4:**
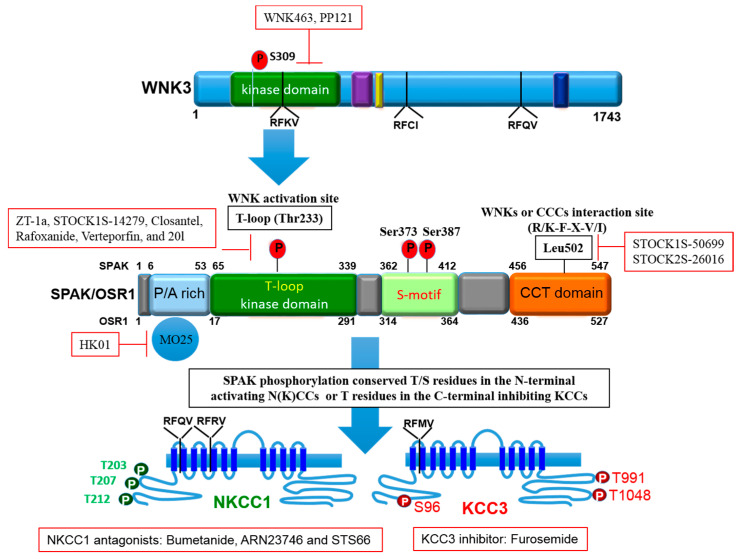
The domain structure of SPAK and the phosphorylation target sites on NKCC1 and KCC3. OSR1 lack the P/A rich (PAPA) domain that is present in SPAK. The figure depicts small molecule inhibitors that target the WNK-SPAK-CCC signaling pathway and their sites of actions. STOCK1S-50699 and STOCK2S-26016 operate through binding to the CCT domain consequently blocking the interaction between SPAK/OSR1 and WNK. STOCK1S-14279, Closantel, Rafoxanide, Verteporfin, and 20l bind the T233E residue on SPAK that is constitutively active or WNK-sensitive. WNK463 and PP121 that inhibit WNKs catalytic activity. HK01, an inhibitor of the mouse protein-25 (M025). Bumetanide, ARN23746 and STS66 are NKCC1 antagonists. Furosemide is a KCC3 inhibitor. ZT-1a is a specific SPAK inhibitor. CCCs, cation-chloride cotransporters; NKCC1, K^+^-Cl^−^ cotransporters; KCC3, K^+^-Cl^−^ cotransporter 3; WNK, with-no-lysine kinase; SPAK, STE20/SPS1-related proline/alanine rich kinase; OSR1, oxidative stress response kinase.

To maintain cell volume homeostasis, the WNK-SPAK/OSR1 kinase pathway activates NKCC1 and simultaneously inhibits KCCs through phosphorylation. Conversely, dephosphorylation inhibits NKCC1 and activates the KCCs [86]. The major phosphorylation sites of NKCC1 include Thr^203^, Thr^207^, and Thr^212^ in the N-terminus whilst the phosphorylation sites of KCC1–4 are located in the C-terminus (Thr^991^ and Thr^1048^ in KCC3 and Thr^906^ and Thr^1007^ in KCC2) [59,72]. Notably, the phosphorylation sites on KCC3, Thr^991^ and Thr^1048^, are conserved amongst all KCC isoforms in humans [72]. Substitution of these threonine residues that make up the sites of regulated phosphorylation inhibited the phosphorylation and subsequent activation of KCC2 and KCC3 [86,87,88]. Recently, it was established that the WNK3-SPAK complex is critical for regulated phosphorylation of KCC3 Thr^991^ and Thr^1048^ residues [86] (also see Figure 3 and Figure 4).

Specific conserved carboxyl-terminal (CCT) domains on SPAK/OSR1 interact with NKCC1 and KCCs [80,86,89]. The Arg-Phe-Xaa-Val/Ile (RFXV/I) domain located in the N-terminal of NKCCs and KCCs is able to recognize the SPAK CCT domain (Figure 3 and Figure 4). Interestingly, a subtype of the KCC2 isoform, KCC2b lacks the RFXV/I motif to facilitate interaction with SPAK. Thus, only KCC2a transport activity decreased when SPAK was overexpressed [53,90]. This interaction of SPAK/OSR1 with both the upstream WNKs and downstream CCCs is crucial for coordinating CCC cellular activity in various osmotic conditions [89,91]. The binding of WNK to SPAK/OSR1 allows for the phosphorylation of residues in the T-loop of the SPAK catalytic domain required for SPAK activation [89,91]. Only once activated is SPAK then able to phosphorylate and inhibit KCC2 and KCC3 at Thr^1048^ and Thr^1007^ respectively and activate NKCC1 at Thr^203^/Thr^207^/Thr^212^. These processes are essential in response to cellular shrinkage and hypertonicity (Figure 1) [53]. In hypertonic conditions, SPAK/OSR1 phosphorylation and activation of NKCC1 is key to achieve RVI [53,67] as an influx of Na^+^, K^+^, Cl^−^ through the NKCC1 along with water will allow for cell volume recovery (Figure 1). Under hypotonic extracellular conditions, water enters the cells and causes cell swelling, subsequently triggering a counter-volume regulation response (RVD). The WNK-SPAK/OSR1 pathway in this condition remains inactive and inhibits NKCC1 activity (Figure 1). Furthermore, the dephosphorylation of KCCs mediated by phosphatase stimulates KCC activity and causes efflux of K^+^ and Cl^−^ along with water, decreasing cell volume (Figure 1) [65]. Thus, pharmacological or genetic antagonistic events of WNK-SPAK/OSR1 will lead to a [Cl^−^]_i_ efflux coupled through simultaneous dephosphorylation of NKCC1 and KCCs. This will then mitigate energy failure occasioned by osmotic stress, as evident in some neurological disturbances such as cerebral edema [32,65,67,86,92].

## 2. Role of NNKCC1 in Stroke

NKCCs play crucial roles in regulating neuronal functions. They are abundantly expressed in neurons throughout the brain and are involved in ion homeostasis maintenance and neuronal excitatory functions [93]. Majorly, they function in regulation and repair of nerve injury through GABAergic signaling [1,94]. However, under specific conditions such as cerebral ischemia, the expression of NKCCs can be altered [94]. Overstimulation of NKCC1 and other major glial ion transporters (such as Na^+^/H^+^, Na^+^/Ca^2+^ and Na^+^/HCO_3_^−^ exchangers) can contribute to glial apoptosis, inflammation, demyelination, inflammation, and excitotoxicity [26]. This cascade of events is involved in the development and progression of neurological diseases such as stroke [26]. Studies have demonstrated evidence of increase NKCC1 expression in neurons, a phenotype resembling immature neurons, following an ischemic stroke [95,96,97]. The altered NKCC1 expression observed post stroke may be responsible for the increased in Na^+^ and Cl^−^ levels in neurons leading to a GABA-mediated depolarization. These events also contribute to a hyper-excitable neuron and cell swelling occasioned by cerebral ischemia [95,98]. Furthermore, disrupted endoplasmic reticulum Ca^2+^ homeostasis [99] and elevated extracellular levels of potassium, glutamate, interleukin-6 [100], interleukin-18 [101], interleukin 1β, and tumor necrotic factor-α [102] which happen during/post cerebral ischemia have been shown to stimulate NKCC1 mRNA gene expression in both neurons and astrocytes. Notably, the elevated extracellular potassium levels seems to be Ca^2+^-dependent as NKCC1 activation is completely terminated either through the removal of extracellular calcium or using Nifedipine to block L-type voltage-dependent calcium channels [103]. Comparatively, similar effects were seen in the expression of NKCC1 mRNA gene in white and gray matter of mutant and wild-type (WT) mice [104]. In addition, [105] an epigenetic study using quantitative real-time RT-PCR technique on cortical slice culture from rats suggested that DNA methylation/demethylation contribute to the regulation of NKCC1 expression during postnatal development and in response to neuronal injury (ischemia) [105].

Following ischemic stroke, both NKCC1 and KCCs are phosphorylated via the WNK-SPAK/OSR1 signaling pathway, leading to NKCC1 activation and KCC inhibition [22,65]. Other contributors leading to NKCC1 activation following an ischemia include: the WNK-calcium binding protein (Cab39; [106]) as well as antagonists of V1 vasopressin [107], MAPK (p38, ERK, JNK, Raf) pathways, cAMP response element-binding protein (CREB) phosphorylation and the ubiquitous transcription factor; hypoxia inducible factor 1-alpha (HIF-1α). This leads to the stimulation of vascular endothelial growth factor (VEGF) expression and ultimate onset of ischemic stroke [37,108,109,110]. Studies on the human subacute ischemic stroke brain tissues demonstrate increased NKCC1 mRNA gene expression [106]. The contribution of NKCC1 protein activation to ischemic brain havocs is now evident as genetic deletion of NKCC1 or its upstream regulator WNK3 in mouse transient middle cerebral artery (MCA) occlusion models displayed minimal infarction, edema, and white matter damage [32,104]. Another study demonstrated increased NKCC1 activity in the perilesional cortex of rats challenged with focal cerebral ischemia induced by endothelin-1 (ET-1) [111]. Furthermore, inhibition of NKCC1 has been reported to reduce edema, Na^+^ uptake, and ischemic injury in rats subjected to STZ-induced hyperglycemic ischemic stroke [112].

## 3. Role of KCC3 in Stroke

Here, we recall as stated earlier in Section 2 of this review that the stimulation/inhibition of NKCCs/KCCs pair via protein phosphorylation is through a reciprocal regulatory mechanism [67,72] (Figure 2). NKCC and KCC participations in cell volume regulations via RVI and RVD mechanisms, respectively, have also been earlier highlighted [19,53,65] (Figure 1). It is only expected that in neuronal functions regulation, activation of KCC3 would play similar physiological roles to those that the inhibition of NKCC1 would. The WNK-Cab39 signaling increased expression of NKCC1 mRNA gene in brain tissues of rats subjected to ischemic stroke. It is proposed to have probable effects on the expression of other cotransporters such as KCC3 [106]. KCC3 expression in the brain requires NKCC1 expression for physiological regulation of cellular homeostasis in the CNS [72,86,87,113,114]. Hence, the roles of WNK-Cab39-KCC signalling in ischemic stroke should be further investigated [106]. In a mouse model study, Lucas et al. [114] demonstrated that alongside inhibited NKCC1, stimulation of KCC3 promoted decreased [Cl^−^]_i_ in the sensory neuron of adult mice. This suggests their involvement in GABAergic/glycinergic transmission as adjudged by its influence on the hyperpolarization of GABA_A_ equilibrium potential (E_GABA-A_) resulting in inhibitory GABAergic neurotransmission due to a decrease in [Cl^−^]_i_. Our recent functional kinomics study alluded that regulatory phosphorylation of KCC3 (Thr^991^/Thr^1048^ residues) is essential for cell volume homeostasis in the mammalian brain [86]. The notion that supports KCC3 physiological role in regulating [Cl^−^]_i_ and consequent influence on GABA polarization state is fascinating and suggests possible relationship between neuronal excitability and cell volume homeostasis [86]. Moreover, this concept behind the physiological function of KCC3 is an indication that it might have a dual role in the regulation of both cell volume and [Cl^−^]_i_ [66] which will be highly relevant in understanding its role in the etiology of stroke.

Furthermore, Byun and Delpire [115] reported that stimulation of KCC3 are involved in cell volume regulation (via RVD) in the nervous system, thereby emphasizing its role in the development and maintenance of myelin and peripheral nerves. The study further established that inhibition of KCC3 by knocking out its expression in mice caused anoxal and periaxonal swelling that ultimately led to neurodegeneration [115]. Another mouse-model study demonstrated that KCC3 gene knockout (KO) in parvalbumin neuron caused peripheral agenesis neuropathy associated with the agenesis of corpus callosum. Similarly, the post-mortem study by Auer and colleagues [116] suggested that neuropathological features observed in the central and peripheral nervous systems (CNS/PNS) could potentially link to genetic defects in axonal KCC3 of CNS/PNS. Indeed, sensory defects in KCC3 knockout (KCC3^−/−^) mice as well as its mutations in humans [63,66,117,118,119] confirm the fundamental role of the cotransporter in peripheral neurons (also see reviews [53,65,66]). Loss of function mutations function of KCC3 have contributed to the pathogenesis of motor and sensory peripheral neuropathy in adult animals and humans [114,115,120,121,122]. Manifestations of peripheral neuropathy or fluid-related axonopathy influence cell volume dysregulation [26,115] and may be involved in the pathogenesis of other neurological conditions such as stroke.

## 4. Role of Regulatory WNK-SPAK/OSR1 Pathway in Stroke

Certainly, the various cellular functional roles of CCCs in the biological system will be compromised without regulatory mechanisms in place. Thus, it is only principally reasonable that the cotransporters actively and continuously maintain their functional integrity through coordinated mechanisms of regulations [53,60,67,69]. Several reports owing to WNK-SPAK/OSR1 kinases as the most involved signaling pathway in the regulation of neuronal Cl^−^ and cell volume homeostasis do exist [60,65,70,71,123] and these established roles of the WNK-SPAK/OSR1-CCC pathway have alluded to their connection with stroke [19,65,71].

There is a growing body of evidence that the WNK-SPAK/OSR1-CCC pathway is involved in pathogenesis of stroke [53,65,70,106,124]. The established roles of the WNK-SPAK-CCC pathway on GABA signaling and cell volume homeostasis are linked to several neurological diseases such as cerebral stroke [53,60,125]. WNK and SPAK/OSR1 kinases are copiously expressed in the CNS [75]. After an ischemic stroke, both NKCC1 and KCCs are phosphorylated via the WNK-SPAK/OSR1 signaling pathway, leading to activation and inhibition of NKCC1 and KCCs, respectively [22,65]. However, inactivating the WNK-SPAK-CCC cascade through concurrent inhibition of NKCC-mediated ionic influx and stimulation of the KCC-mediated ion efflux has been shown to reduce cellular swelling in ischemic stroke brains [53,65]. The regulatory role of WNK-SPAK-CCC in cellular ionic homeostasis have also been shown to contribute to post-ischemic stroke infarction and cerebral edema [66]. Thus, inactivation of the WNK-SPAK-CCC cascade would trigger the simultaneous inhibition of NKCC mediated ionic import and stimulation of KCC mediated ionic export to eradicate cellular osmotic imbalance [53,65]. It has also been reported that estradiol increases NKCC1 phosphorylation consequently promoting GABA-mediated depolarization [126]. This occurs through stimulation of SPAK and OSR1 that is transcription dependent [127]. Studies using focal ischemia rat model have shown that estradiol treatment promotes neurogenesis in the subventricular zone of the brain, probably by increased expression of HIF-1α and VEGF [128]. WNK phosphorylate SPAK/OSR1, which in turn, phosphorylate NKCC1 and KCC3 at key regulatory sites [129]. Previous reports have shown that SPAK has a CCT domain to interact with NKCC1 and the KCCs [11,89,91,130]. However, the understanding of their physiological functions in normal and ischemic brains are still elusive [92].

Indeed, WNK isoforms are selectively expressed in the CNS [131] and WNK3 is mostly expressed in the brain [132]. This particular WNK isoform exerts its action on NKCCs and KCCs reciprocally [53,113]. Thus, the reciprocal actions of WNK3 on NKCC1 and the KCCs along with its concurrent expression with cotransporters in GABAergic neurotransmission that undergo dynamic changes in [Cl^−^]_i_, suggest its involvement in regulation of neuronal CCCs [53,133,134]. In fact, Kahle et al. [113] provided a compendium of data that suggested WNK3 as a dynamic regulator of NKCC1 and KCCs physiological activities. Simultaneous expression of WNK3 and NKCC1 in neurons may lead to enhanced phosphorylation of regulatory sites in NKCC1 and a consequent increase in the activity of NKCC1 [32,135]. The target of protein phosphatase 1 (which recognizes the consensus motif: RVNFXD) is a highly conserved RVNFVD sequence that is located in the amino-terminus of NKCC1. The RVNF binding motif overlaps with the SPAK binding motif (RFRV). A slight mutation of this sequence will cause NKCC1 activity to increase [136]. Interestingly, phenotypes of NKCC1 inhibition and KCC activation due to inactive WNK3 signaling pathway are reversed by potential protein phosphatase 1 inhibitors such as calyculin A and cyclosporine A [133,137]. According to Melo et al. [138], WNK3 inhibits the activity of KCC3 by promoting the phosphorylation of Thr^991^ and Thr^1048^ as well as Ser^96^, a third phospho-site involved in KCC3 regulation (also see Figure 2). Double (KCC3-T991A/T1048A) or triple (KCC3-S96A/T991A/T1048A) alanine mutations of KCC3, activated the cotransporter, which further increased hypotonicity. Thus, the study suggested that the phosphorylation of WNK3 signaling pathway was disabled, subsequently activating KCC3 by cell swelling [138].

Certainly, the upstream WNK3-SPAK/OSR1 pathway regulation of NKCC1 activity coupled with inhibition of KCC3 is implicated in the pathology of ischemic stroke [86]. Previous studies have demonstrated that WNK3 KO mice exhibited a reduction in infarct volume and axonal demyelination coupled with diminished cerebral edema and improved neurological behaviors following cerebral stroke when compared to WT mice with significantly activated WNK3 [32,86,92]. However, it is important to note that WT mice showed better survival and functional outcomes after a brain edema in comparison to mouse models lacking aquaporin-4 (AQP4) [139], a water transport system that allows for bidirectional water flux. As such, further research will need to elucidate the distinction between the role of AQP4 and WNK3 in cerebral edema and stroke. Thus, Begum and colleagues [32] observed stimulation of WNK3 and SPAK kinases in cortical neurons and primary oligodendrocytes cultured from the brain of mice subjected to transient MCA stroke [32]. They further established that cerebral ischemia facilitates hyper-phosphorylation of the WNK3-SPAK/OSR1 catalytic T-loop and of NKCC1 stimulatory sites (Thr^203^/Thr^207^/Thr^212^); thus, increased expression of NKCC1 in the brain cells [32]. However, transgenic KO of WNK3, abridged ischemia-mediated SPAK/OSR1-NKCC1 phosphorylation and displayed reduced cerebral edema, axonal demyelination, and infarct volume, as well as improved post-stroke neurological recovery when compared to WT mice [32]. Briefly, the data presented by Begum et al. [32] identify the role of WNK3-SPAK/OSR1-NKCC1 signaling pathway in ischemic neuroglial injury and suggested that obstruction of this pathway could reduce NKCC1 expression in the brain and avert post-stroke neuronal cell death following [32]. Similarly, Zhao et al. [92] demonstrated that KO of the WNK3-SPAK kinase complex in mice instigates decreased expression of NKCC1 and subsequently ameliorated cerebral infarction and edema after MCA stroke. Generally, deletion of the WNK3-SPAK kinase complex significantly produced less cytotoxic edema, less demyelination, and improved post-ischemic stroke neurological outcomes in the transgenic mice [92]. However, it is worth noting that the mechanism(s) of regulations employed by WNK3-SPAK/OSR1-NKCC1 signaling pathway in oligodendrogenesis is still elusive and requires further studies [26]. In addition, we recently demonstrated that WNK3 KO mice exhibit reduced endothelial and perivascular cytotoxic edema of astrocytes following post-ischemic stroke [86]. We further alluded that WNK3-SPAK inhibition confer neuroprotection on mammalian brain through concurrent stimulation of KCC3 activity at Thr^991^ and Thr^1048^ residues and inhibition of NKCC1 activity at Thr^203^, Thr^207^, and Thr^212^ residues [86].

In a recent rat model study, Bhuiyan et al. [106] demonstrated that WNK-Cab39-NKCC1 signaling pathway is implicated in ischemia. Furthermore, they suggested that activated WNK-Cab39 pathway increased NKCC1 activity in brain tissues of spontaneous hypertensive rats following subacute ischemic stroke [106]. A more recent similar report by Huang et al. [124] demonstrated that ischemic stroke with hypertension comorbidity further stimulates the WNK-SPAK/OSR1-NKCC1 signaling pathway, which contributes to deteriorated neurological functions/behavior [124]. In fact, the established role of WNK-SPAK/OSR1 signaling pathway in stimulating NKCC1 and inhibiting KCC3, which contribute to the pathogenesis of stroke, are the reasons for our recent pharmacological studies [70,71].

## 5. Current Pharmacological Treatments for Stroke

We underscored in the earliest section of this review that stroke is one of the major threats to global health. Over the years, stroke had been a chief contributor to mortality and disabled lives across the globe; there are projections that the impact of this disease on global health may be worse in the near future. Presently, there are only few pharmacological strategies available to reduce the health and socio-economic burden triggered by this disease. Thus, there is an urgent need to tackle this disease. In this regard, research on the role of CCCs in the pathogenesis of stroke to inform future drug development is needed. Accordingly, this section of the review highlights current pharmacological approaches in the management of stroke with particular focus on molecular compounds that potentially inhibit SPAK/OSR1 pathway and NKCC1 and stimulate KCC3.

### 5.1. Inhibitors of WNK-SPAK/OSR1 Pathway

A quick recap of the following crucial information from previous sections, which may contain pharmacological strategies for managing stroke: (1) WNK-SPAK/OSR1 modulates the activities of CCCs through a well-coordinated reciprocal pattern of regulation [53,60,67,69], (2) activation of WNK-SPAK/OSR1 signaling pathway stimulates NKCC1 and inhibits KCC3 expressions [19,53,67,72], and (3) development and progression of stroke have been implicated with phosphorylated WNK-SPAK/OSR1 signaling pathway and subsequent up- and down-regulations of NKCC1 and KCC3 expressions, respectively [22,140]. Therefore, we can safely presume that molecular compounds that act as pharmacological or genetic antagonists of WNK-SPAK/OSR1 kinases are likely potential drug candidates for the treatment of stroke. Recent reports have shown that drugs that are potent blockers of WNK-SPAK/OSR1 signaling pathway reduce phosphorylation of NKCC1 and KCC1 which enables cellular chloride expulsion, subsequently mitigating cerebral edema and other neurological anomalies following ischemia. This then protects against brain damage and enhances post-stroke brain functions [32,65,70,86,92,94,124,141].

The WNK-SPAK/OSR1 pathway constitutes potential therapeutic targets in Cl^−^ dysregulation [23]. The loop diuretic, bumetanide protects the brain from damage by mediating GABAergic signaling in NKCC1 expression following ischemic injury [22,23,26,142]. Furthermore, treatment with the drug reversed the impact of GABA-mediated depolarization, which may promote functional recovery after stroke via neuron repair/protection as adjudged by its effect of GABA_A_ receptor antagonist and WNK3 knockout [94,142]. Pharmacologically targeting the WNK-SPAK/OSR1 kinase pathway could be a strategy to restore GABAergic inhibition [23]. Indeed, genetic or pharmacological inhibition of WNK-SPAK/OSR1 activity would lead to cotransporter dephosphorylation: inhibition of NKCC1 and activation KCC3, which would enhance [Cl^−^]_i_ extrusion [67]. Furthermore, enhancement of [Cl^−^]_i_ extrusion in the neurons would facilitate GABA_A_ receptor-mediated hyperpolarization and thus inhibit neuronal activity through combined NKCC1 inhibition and KCC3 stimulation [19,22,53,65,67] (Figure 2).

Recent studies have indicated that decline in NKCC1 protein expression along with WNK3 knockdown, contributes to lessened post-stroke brain injury and accelerated neurobehavioral recovery [32,86,92]. These reports and more have immensely motivated the development of novel therapeutic strategies that have targeted WNK-SPAK/OSR1 signaling pathways to improve post-stroke physiological functions [70,106,124]. A recent rat model study demonstrated an upregulation of WNK-SPAK/OSR1-NKCC1 signaling pathway in the brains of spontaneously induced-hypertensive rats and subsequently augmented susceptibility to ischemic damage [106]. However, intraperitoneal administration of bumetanide (10 mg/kg) post-reperfusion blocked the WNK-Cab39-NKCC1 signaling pathway and subsequently mitigated post-ischemic infarction and cell swelling and improved neurological functions in animals [106]. Loop diuretics are often used to inhibit NKCCs. Inhibition of NKCC2 promote diuresis in the kidney and reduces pressure due to excess fluid in the lungs. Hence, loop diuretics are a treatment option hypertension and pulmonary edema. Although some loop diuretics inhibit KCCs, they do so very poorly. Researchers have explored the loop diuretics bumetanide and furosemide as novel treatment options for brain disorders [65]. However, a number of unfavorable physiochemical characteristics associated with the use of bumetanide [23,41,65] call for better alternatives in the management of neurological diseases including stroke. Recently, Huang and co-workers [124] reported that a novel NKCC1 inhibitor (STS66) is superior to bumetanide in ameliorating ischemic brain injury following transient MCA occlusion and large-vessel ischemic stroke models. In the study, ischemic injury stimulated WNK-SPAK-NKCC1 cascades in brains of AngiotensinII (AngII)-induced hypertensive mice. However, STS66 treatment completely blocked this pathway and by implication mitigated ischemic infarction, cerebral edema, and neuronal death as well as neurological deficits in both stroke models with hypertension comorbidity [124].

We recently proposed that improved understanding of cooperative interactions among different phospho-sites of cotransporters and the molecular mechanisms involved in their physiological regulations could provide insights to inform potential pharmacological interventions [71]. Recently, we conducted a large-scale phospho-proteomics study with the application of immunoblot and phospho-antibodies immunoprecipitation techniques to investigate the regulatory mechanisms of a broad kinase inhibitor, staurosporine and N-ethylmalemide (NEM), a modulator of both kinase and phosphatase activities on phosphorylation of specific KCC2 and NKCC1 in HEK293 cells and immature cultured hippocampal neurons [71]. Our analyses revealed dephosphorylation of Thr^203^, Thr^207^, and Thr^212^ of NKCC1 and Thr^1007^ of KCC2 following application of the two agents. The two compounds resulted in dephosphorylation of sites Thr^233^ and Ser^373^, phosphorylation sites located within the T-loop and S-loop of SPAK. Hence, the study suggests the inhibitory effect of staurosporine and NEM on WNK-SPAK/OSR1 signaling pathway in the regulation of NKCC1 and KCC2 is in a reciprocal pattern [71]. We are of the opinion that the underlying information from this study will be highly important for future development of integrative therapeutic strategies in the management of neurological diseases such as cerebral stroke.

Importantly, evolving roles of WNK-SPAK/OSR1 signaling in stroke as discussed in this review, points to additional possible applications of WNK-SPAK/OSR1 modulation in neurological diseases. In view of this, a promising strategy could involve exploitation of the unique structure of these kinases to enhance protein specificity [53,70]. Immense efforts to inhibit WNKs or SPAK/OSR1 for the treatment of human diseases such as hypertension have led to the discoveries of small molecule inhibitors. WNK kinase inhibitors include WNK463 [143], PP121 [144], and SPAK inhibitors such as STOCK1S-14279, Closantel [145], Rafoxanide [146], Verteporfin [74], STOCK1S-50699, and STOCK2S-26016, [147], as well as HK01 [148] and 20I [149] (also see Figure 3 and Figure 4). Unfortunately, none of these compounds is an ideal drug candidate for the treatment of brain disorders due to their relatively low penetrability through the blood-brain barrier (BBB). Recently, we employed a scaffold-hybrid strategy in our laboratory to develop a novel compound ZT-1a. ZT-1a is a non-ATP-competitive SPAK blocker, which specifically inhibits this signaling pathway by decreasing SPAK-dependent phosphorylation of NKCC1 and KCCs in cell cultures as well as in vivo mouse and rat brains [70]. In brief, treatment with ZT-1a (2.5–5.0 mg/kg) abated post-stroke related brain injuries and improved neurological features/functions. The data from the study suggests that ZT-1a or related compounds that are CCC modulators could be a therapeutic strategy for neurodegenerative disorders such as cerebral stroke [70]. Hence, we holistically advocate for follow-up with detailed research studies on the development of more WNK-SPAK/OSR1 inhibitors with favorable pharmacokinetic properties for clinical use.

### 5.2. Inhibitors of NKCC1

It has been established that the recovery process from many neurological disorders including stroke would highly benefit from inhibition of NKCC1 activity [94]. Suppression of NKCC1 activity through bumetanide is neuroprotective and improves post-stroke neurophysiological status [142,150]. Thus, bumetanide has the potential to influence many CNS disorders [94]. Several studies have demonstrated the contribution of NKCC1 in the development and progression of post-stroke edema and cell death, thus targeting NKCC1 could be a potential neuroprotective target [22,26,32,65,111,151]. In fact, a pharmacological study using bumetanide demonstrated a significantly reduced neuronal Na^+^ overload and cell death. Bumetanide also simultaneously reduced infarct volume and brain edema [104]. Another rat model study showed that bumetanide administered after focal cerebral ischemia in rats (given 7 days post-ischemia, and continued for 21 days), improved behavioral recovery and promoted neurogenesis 4 weeks post-havoc [141]. Low concentrations of bumetanide (2 to 10 μM) are capable of inhibiting NKCCs in vitro with no significant effect on the KCCs; a high concentration has been shown to inhibit the activities of both NKCC1 and KCC2 [152,153]. The expression of NKCC1 is common at the luminal side of endothelial cells of the BBB, thereby allowing easy interaction between the transporter and its inhibitor (bumetanide) when administered intravenously, which subsequently decreased edema in MCA occlusion model of stroke in rats [154]. Bumetanide acts through docking to the binding site at the trans-membrane region of NKCC1. Docking to this region allows for the inhibition of NKCC1 activity and reduced [Cl^−^]_i_ in neurons [94]; a more likely mechanism through which the drug confers neuroprotection and neuronal recovery following stroke episodes [94,155].

Simard et al. [156] and Walcott et al. [42] in their respective reviews highlighted the implication of the constitutive expression of NKCC1 and SUR1-regulated NC_Ca-ATP_ (SUR1/TRPM4) channel on the cascade of events that are involved in the pathogenesis of cerebral ischemia and the impact of combinatorial therapy of bumetanide and glibenclamide in ameliorating the havoc. Bhuiyan and colleagues [106] reported that bumetanide downregulated the WNK-Cab39-NKCC1 signaling pathway, consequently reducing the susceptibility of hypertensive rats to ischemic brain damage. Furthermore, in a recent study a synergistic treatment with mild hypothermia (33.5 °C for 30 min) and inhibitor DAPT (50 µM) attenuated the overexpression of NKCC1 mRNA following global cerebral ischemia injury in rats [20].

In animal stroke models, bumetanide administration pre- and post-stroke induction led to the down-regulation of NKCC1 expression. Other observations include a reduction in edema, infarction volume, and ischemic necrotic cell death especially in the early stage of ischemic damage, promotion of neurogenesis, and improved sensorimotor recovery [17,97,109,141,157,158]. In another rat model study, ET-1 was used to induce focal ischemia but post treatment with bumetanide selectively inhibited NKCC1 expression in the cortex and promoted synaptic plasticity in the denervated cervical spinal cord following cerebral ischemia [111]. Similarly, Xu et al. [141] demonstrated that chronic treatment with bumetanide promotes neurogenesis and behavioral recovery after ET-1-induced stroke in rats. In addition, bumetanide (10 µM) was used in another study to block NKCC1 in order to facilitate decreased [Cl^−^]_i_ in hippocampal tissue cultured from rats either during oxygen-glucose deprivation for 120 min or post-exposure. The drug improved neuronal viability during the acute ischemic episode which suggested its critical role in the modulation of transmembrane chloride transport [27].

Indeed, bumetanide appears to be a promising pharmacological inhibitor of NKCC1; it possess some demerits that may limit its application as an anti-stroke drug to some extent [23,94]. Alongside bumetanide, a novel inhibitor STS66 (a prodrug of bumetanide) also exhibits promising potential as a pharmacological inhibition of NKCC1 and has been demonstrated to also reduced ischemic infarction, swelling and neurological deficits in mice model of transient ischemic stroke [124]. Interestingly, STS66 can penetrate BBB more easily and appears to be more efficient in eliciting the aforementioned anti-stroke properties [124], which is one of the various reasons it has been recently proposed as a better therapeutic drug in stroke management when compared with bumetanide [23,65]. A finding contrary to the common hypothesis on the efficacy of bumetanide was recently reported [18]. In this study, post treatment with bumetanide (40 mg/kg) following Intracerebral hemorrhage induction in male Sprague Dawley rats failed to improve behavior or lessen injury neither did the drug normalized ion concentrations after late dosing [18].

In spite of the positive outcomes demonstrated clinically by administration of bumetanide to patients with psychiatric/neurological conditions [159,160,161,162,163,164,165,166]; the drug has exhibited strong diuretic effect resulting from the inhibition of NKCC2 expression in the kidney which may pose serious challenges to issues on drug compliance and health concerns [167,168,169], thereby limiting the therapeutic applications of bumetanide. Hence, selective inhibition of NKCC1 in lieu of renal NKCC2 may attenuate the diuretic glitches. In this regard, Savardi et al. [170] recently discovered ARN23746, a selective inhibitor of NKCC1 in lieu of NKCC2 and KCC2 in vivo. The reports from the study demonstrated that the pharmacokinetic profile of ARN23746 is better when compared with that of bumetanide in vitro and in vivo. Briefly, the study demonstrated that ARN23746 (10 μM) restored aberrantly high [Cl^−^]_i_ to the physiological level in mature hippocampal neuronal cultures of Ts65Dn mouse model Down syndrome (DS) coupled with rescued cognitive impairment in Ts65Dn with no significant diuretic effect in either the WT or Ts65Dn mice [170]. Furthermore, the researchers demonstrated that intraperitoneal administration of ARN23746 recovered social and repetitive behaviors associated with the main symptoms of autism spectrum disorder (ASD) in valproic acid (VPA) mouse model of ASD. In addition, neither diuretic effect nor overt toxicity of the compound were present in the ARN23746 treated mice [170]. ARN23746 has great potential for further development into a clinically-relevant drug for the treatment of DS, ASD [170], and possibly several other neurological conditions characterized by impaired Cl^−^ homeostasis including stroke.

### 5.3. Activator of KCC3

The KCCs, especially KCC2 and KCC3, are popular due to increased findings on human disease-causing mutations [68,121,122]. Hence, the discovery of small molecules that modulate these cotransporters’ activities is prioritized within the field. Discovery of such modulators may aid development of therapeutic drugs for the management of KCC-related diseases as well as other pathological conditions including stroke [171]. Currently, the loop diuretics bumetanide and furosemide are the only FDA-approved drugs that modulate the KCCs [172]. In a mouse model study, bumetanide is demonstrated to be involved in the stimulation of KCC3 expression and subsequent extrusion of [Cl^−^]_i_ in the sensory neurons [114]. In addition, Adragna and co-workers [87] in a cell culture study substituted Thr^991^ and Thr^1048^ residues with alanine at the carboxyl terminus of KCC3a protein, which prevented inhibitory phosphorylation at the substituted sites and subsequently triggered increased expression of KCC3a mRNA. Interestingly, the flux condition accompanied a down-regulation of NKCC1 expression, facilitated by the addition of ouabain (0.1 mM), and bumetanide (10 μM) to the flux media [87].

However, Delpire and Weaver [171] recently expressed their concerns for the need to develop modulators of KCC activity to provide insights into KCC modulation as a therapeutic strategy for neurological conditions such as stroke. Unfortunately, these FDA approved drugs (bumetanide and furosemide) are poor inhibitors of KCCs, with a higher potency for NKCC1 or NKCC2 (IC_50_ = 0.5–5.0 μM) in comparison to KCC (IC_50_ = 50–500 mM) [33,171]. In fact, drugs that can act as weak inhibitors might be better alternatives as complete inhibition mimics a loss-of-function, which could presumably be harmful for the nervous system. As the KCC isoforms have different expression patterns and physiological functions, target specificity in the deployed pharmacological approach is also an issue [172]. In a large screening effort targeted against KCC2, Delpire et al. [173] was able to identify inhibitory compounds more potent (3–4x) than the two loop diuretics. However, these compounds are not ideal drug candidates due to the following reasons: (1) non-specificity to KCC2 as they concurrently inhibit KCC3, and (2) poor pharmacokinetic properties [173,174]. Meanwhile, the ability of loop diuretics to reach the CNS/PNS remains obscure [171,172]. Perhaps a better pharmacological approach would be to develop therapeutic compounds that are specific modulators of the KCC3. Fortunately, our recently developed novel molecular compound, ZT-1a, is a SPAK kinase inhibitor that specifically stimulates KCC3 and inhibits NKCC1 by decreasing their SPAK-dependent phosphorylation/signaling pathway in cultured cells and in vivo rat and mouse brains [70] (also see Figure 2). In addition, the systematic administration of ZT-1a ameliorated phosphorylation of co-transporters and cerebral edema following ischemia, protect against brain damage and improve neurological functions after stroke episode [70].

## 6. Conclusions and Future Directions

The CCCs play crucial roles in regulating neuronal functions. The cotransporters are key mediators of several and important cellular functions such as cell volume regulation, trans-epithelial ion transport, and maintenance of [Cl^−^]_i_. Modulation of NKCC1 and KCC3 expressions by their upstream regulator, WNK-SPAK/OSR1 is implicated in the development and progression of stroke. There are several demonstrations that phosphorylation of NKCC1 and KCC3 via the WNK-SPAK/OSR1 signaling may lead to activation of NKCC1 and inhibition of KCC3 either during or post-stroke episode. In fact, the role of NKCC1 and KCC3 as well as their regulatory proteins in stroke pathogenesis suggests that they are potential targets for the treatment of stroke. The pharmacological strategies that were discussed in this review possess potential therapeutic efficacies for stroke management. Novel compounds must successfully address concerns regarding off-target effects due to the many isoforms and physiological function related to the WNK-SPAK/OSR1-CCC pathway. As advances in stroke therapy may also benefit other neurological impairments, we strongly suggest consistent follow-up actions on currently available pharmacological treatments for stroke through detailed research studies to aid further development of therapeutic drugs with a better pharmacokinetic profile. Hence, we holistically advocate for increased focus on human clinical research on this topic as informed by its paucity to that regards.

## Data Availability

Not applicable.
